# Progress in Using Systematic Reviews of Animal Studies to Improve Translational Research

**DOI:** 10.1371/journal.pmed.1001482

**Published:** 2013-07-16

**Authors:** C. R. Hooijmans, M. Ritskes-Hoitinga

**Affiliations:** Radboud University Nijmegen Medical Centre, SYRCLE at Central Animal Laboratory, Nijmegen, The Netherlands

## Abstract

Carlijn Hooijmans and colleagues discuss developments that might improve the quality and translation of animal research, focusing on the importance of systematic reviews, the role of an international register of animal studies, and cooperation across the scientific community.

*Please see later in the article for the Editors' Summary*

**Summary Points**

- During the last decade, new developments and initiatives have been introduced to improve the quality and translational value of animal research. We summarize these here, focusing on quality of study conduct, reporting, and replication; systematic reviews and meta-analyses; and study registration, publication bias, and data sharing.
- Systematic reviews of animal studies should be conducted routinely. Funding agencies should subsidize systematic reviews, not simply for transparency, but also to avoid waste of financial resources and unnecessary duplication of animal studies.
- An international register for animal studies should be established and funded. Journals should publish “negative and neutral” results, and promote data sharing.
- Improving the quality and translation of animal research requires co-operation from the wider scientific community, journals, researchers, regulators, funding bodies, peer reviewers, and patients. Systematic reviews of preclinical studies should be included in the Cochrane Library.

## Introduction

The scientific rationale for systematic reviews of animal studies was first outlined in an influential commentary published in 2002 [1]. The authors drew attention to the need for more rigorous assessment of animal studies before beginning studies in patients. They suggested that systematic reviews of all relevant animal experiments should be a prerequisite for the design of new clinical trials. Their concerns were prompted by a systematic review of animal studies of Nimodipine in focal cerebral ischemia, which showed insufficient evidence to justify involvement of over 7,000 patients in drug trials [2].

We summarize developments since 2002, focusing on the scientific rationale for systematic reviews of animal studies and the limitations and pitfalls. Moreover, we suggest further improvements in animal research to maximise its contribution to evidence-based translational research.

## Translational Challenges

Although it may be preferable to study humans when seeking knowledge about human biology or the human response to interventions, it may be unethical or unfeasible. In these circumstances animal models are regularly used. One of the advantages of animal studies is the ability to study a relatively homogeneous group of animals instead of a heterogeneous group of patients. In addition, animal studies offer a wider range of possibilities to examine toxicity of interventions or study pathology and mechanisms of disease; most clinical trials only focus on clinical efficacy. Nevertheless, new therapies or interventions shown to be effective in animal studies are often less effective or ineffective in clinical trials [3]. Sometimes, interventions are even harmful to humans [2],[4],[5].

Several challenges exist to successfully translating the outcomes from animal research to humans in a clinical setting:

(1) Biological differences between species and strains

Genetic and species differences between animals and humans, but also within animal species, strains, and cell lines are often disregarded in the design of animal studies [6]–[8]. Ignoring biological differences between species and strains results in flawed design and unreliable outcomes, incurs unnecessary costs, and uses more experimental animals than necessary.

(2) Poor methodological quality of animal experiments

In many animal experiments, important methodological issues, such as randomization and blinding, are neglected [9]–[12]. In addition, the statistical methods used to analyze results are often flawed [11]. These failures mean that basic research cannot be replicated and may cause an overestimation of the efficacy of interventions [13]. Although clinical trials in humans also suffer from biases, preclinical animal studies appear to be associated with even greater risks [14].

(3) Differences in the design of experimental animal studies and clinical trials

Animal studies designed to decide whether or not to take an intervention forward to clinical trials, use study protocols that differ from clinical studies. For example, many of the animal studies investigating the effect of probiotics on pancreatitis administered probiotics *before* inducing pancreatitis, whereas in the clinical trial probiotics were given to patients already presenting with signs of pancreatitis [5].

(4) Insufficient reporting of details of animals, methods, and materials

Characteristics of the design of animal studies, such as the strain, gender, age and weight, and housing conditions of the animals used, are known to influence results. Failures in reporting these details skew the interpretation of study results and subsequent translation into clinical benefits.

(5) Publication bias

Not reporting experiments with negative or neutral results leads to an overestimation of the effect of an intervention [15]. Publication bias plays a role in both clinical trials and animal studies, but is believed to be more problematic in animal studies (14). In experimental stroke studies, for example, an estimated 14% of animal studies are unreported [16].

## Scientific Rationale for Systematic Reviews of Animal Studies

In light of these translational challenges, it is no surprise that results in clinical trials often deviate from results of animal studies [17]. Systematic reviews are an important part of the solution because of the need to better understand how animal research informs clinical research [18]–[22].

By systematically reviewing the literature and ensuring that all animal studies are published regardless of outcome, unnecessary duplication of often expensive animal experiments can be reduced [23]. Moreover, systematic reviews can contribute to improving translation of animal research to humans in several ways: information about safety and efficacy of treatments that is hard to obtain from individual studies; misinterpretation of evidence is diminished because systematic reviews expose biases and inadequacies in the methodology of individual studies [24]; and differences in design between animal studies, and between animal and human studies, become transparent, and the most optimal animal models for evaluating a treatment before testing in humans can be selected [25],[26].

Systematic reviews of animal studies also have limitations. First, reviews are time consuming. Second, because of poor reporting and methodological inadequacies in animal studies, reviews may be unable to produce precise and reliable overall effect estimates. For now, systematic reviews should be primarily used to determine the direction of the effect, and the factors affecting the size and direction of the effect. Third, systematic reviews will be unable to address the issue of the low external validity of (certain) animal models to the extent that this is due to biological differences between humans and other animal species. [27]. Nevertheless, overall, the advantages of systematic reviews outweigh the limitations.

## Improving the Translational Value of Animal Studies

Since 2002, when the scientific rationale for systematic reviews of animal studies was published [1], important developments and initiatives have aimed to improve the quality and translational value of animal research:

(i) Quality of study conduct, reporting, and replication

In 2009, the NC3Rs surveyed the quality of reporting, experimental design, and statistical analysis in 271 published animal studies [11]. The survey revealed a number of weaknesses in experimental design, statistical analysis, and reporting, and prompted publication of two sets of guidelines. The Animal Research: Reporting In Vivo Experiments (ARRIVE) guidelines [28] and the Gold Standard Publication Checklist (GSPC) [29] are based on the CONSORT guidelines for reporting clinical trials, and offer checklists for improving reporting of animal research. These initiatives may help to reduce bias in the conduct and reporting of animal studies, and thereby improve their predictive value [12]. Major scientific and medical journals have included the ARRIVE guidelines in their guidance to authors, and The Netherlands Organisation for Health Research and Development (ZonMW) urges researchers who receive funding from this organisation to use the GSPC. Moreover, in the UK, the Wellcome Trust, the Biotechnology and Biological Sciences Research Council, and the Medical Research Council have signed up to the ARRIVE guidelines. ZonMW has also issued a special funding programme for further development of education and supervision in this field.

Furthermore, an online interactive educational programme, known as 3Rs-REDUCTION (http://www.3rs-reduction.co.uk/) has been developed for researchers to improve the design of animal experiments and to better consider genetic variation in animal (toxicology) testing. Workshops arranged by the Fund for the Replacement of Animals in Medical Experiments (FRAME) complement this programme, and aim to educate animal researchers in methods and practices to use the minimum number of animals necessary for achieving the objectives of a study.

A key feature of good experimental practice is reproducibility. Reproducibility, or the lack of it, is a familiar issue for the scientific community, and the Science Exchange, *PLOS ONE*, Figshare, and Mendeley have recently launched the Reproducibility Initiative (https://www.scienceexchange.com/reproducibility).

This initiative aims to identify and reward high quality, reproducible research. Results can be submitted for independent and “blinded” validation. Validated studies receive a “Certificate of Reproducibility,” acknowledging that their results have been independently reproduced as part of the Reproducibility Initiative. Scientists are able to publish the replicated results as an independent publication in the *PLOS ONE* Reproducibility Collection, and can share their data using the Figshare Reproducibility Collection repository. The director of the US National Office of Public Health Genomics says that the initiative “will begin to address huge gaps in the first of many translational steps from scientific discoveries to improving health.”

(ii) Systematic reviews and meta-analyses

Other initiatives are directly focused on the conduct of systematic reviews and meta-analysis of animal studies. In 2004, Pound and colleagues [30] called for a large-scale programme of systematic reviews of animal studies to help improve the quality of evidence derived from animal data. That same year, the chair of the National Institute for Clinical Excellence called for “detailed scrutiny of the totality of all the available evidence” to assess the “real predictive power” of the preclinical biological studies used in the research and development process [31].

In response to these appeals, an international group of animal researchers established the Collaborative Approach to Meta Analysis and Review of Animal Data from Experimental Studies (CAMARADES). This group focused particularly on systematic reviews of animal studies evaluating interventions for stroke and other neurological conditions.

In 2005, the Nuffield Council on Bioethics published a report stressing that more systematic reviews of animal studies are needed to understand the ethical and scientific issues in animal research [32], and urged funders of animal research to support systematic reviews. Simultaneously, the American Council on Science and Health called on US government agencies to adopt new guidelines for toxicology studies to identify cancer-inducing substances. The council insisted that modern methods be used to review systematically “the totality of evidence from animal studies, just as is done for studies on humans, rather than giving excessive weight to any one or two animal test results in one species” [33]. At the same time, a British charity, SABRE Research UK, was established by patients and researchers to promote systematic reviews of animal studies for better health care, and to protect patients and research volunteers from unsound research.

In 2008, the SYstematic Review Centre for Laboratory animal Experimentation (SYRCLE, previously named 3R Research Centre) was established in Nijmegen (The Netherlands) to improve the scientific quality and transparency of animal research, and to develop educational material and guidelines for systematic reviews of animal studies. In 2012, the Dutch Parliament adopted a motion in which the government was requested to ensure that systematic reviews also become the norm for animal experiments, just as is the case for clinical studies.

Although it is too early to provide evidence that systematic reviews lead to improved translational value of animal research, there are some examples [3],[34],[35]. The systematic review of Hirst et al., which was conducted after establishment of temozolomide as first line chemotherapy for malignant glioma, showed that temozolomide, also improved survival and reduced tumour volume in experimental glioma, just like in humans, even after accounting for publication bias [34]. In addition, the design of an ongoing trial on hypothermia in acute ischemic stroke (EuroHYP-1) was explicitly based on a systematic review of animal data [35]. Evidence-based selection of animal models will also improve translation, as the use of less suitable animal models is decreased [8],[25].

Scientists are increasingly convinced of the need of systematic reviews of animal studies. The number of systematic reviews of animal studies increased from 86 to 244 between 2005 and 2010. However, systematic reviews of animal studies are still relatively rare [15].

(iii) Study registration, publication bias, and data sharing

Obtaining data originating from preclinical animal studies is a priority, and calls have been made for prospective registration of all animal experiments [3],[24]. Although a registration system does not currently exist, initiatives have begun. The Dutch parliament adopted a second motion in February 2012 that defined a general database/registry of animal studies with the aim of preventing unnecessary duplication and reducing publication bias. The Netherlands Knowledge Centre on Alternatives to animal use (NKCA) is tasked with outlining the steps needed to implement this motion. A register of animal studies has different pitfalls and challenges compared to a registry of clinical studies.

Another strategy for obtaining data originating from preclinical animal studies, is publishing negative and neutral results, and promoting data sharing. In 2012, Begley and Ellis called for opportunities to present negative data, and suggested that journal editors should initiate this cultural change [36]. In addition, funding agencies and reviewers must acknowledge that negative data are just as useful as positive data. The Dutch ZonMw recently developed a special funding programme for animal research entitled, “More knowledge with fewer animals,” including a module providing funding to scientists for publishing negative or neutral results.

Several initiatives aimed at data sharing are being developed. First, REACH, the European Community Regulation on chemicals and their safe use (EC 1907/2006) (http://echa.europa.eu/chemicals-in-our-life/animal-testing-under-reach) encourages information from tests to establish that the hazardous properties of chemicals are shared between registrants. Sharing of results of tests in vertebrate animals is already mandatory. The European Chemicals Agency, ECHA, facilitates sharing of data and information by companies, scientists, and citizens.

Second, Figshare was launched in 2011. Figshare allows researchers to publish research findings in an easily citable, sharable, and discoverable manner. By opening up the peer review process, researchers can publish neutral results, avoid the file drawer effect, and help make scientific research more efficient. Figshare uses creative commons licensing to share research data and allow users to retain ownership.

Third, *F1000Research*, the first open science journal, was launched in 2012. It is an open access journal for life scientists, offering immediate publication, transparent post-publication peer review, and full data deposition and sharing. All scientifically sound articles are accepted. This revolutionary development ensures and stimulates full openness and transparency.

## Future Horizons

Although these initiatives are promising, there is no room for complacency. We must start by converting ideas into daily scientific practice.

More systematic reviews of animal studies are needed. Making systematic reviews of animal studies a routine is our scientific and societal responsibility, just as with clinical studies in humans. As a minimum, we urge researchers to conduct a systematic search for all relevant experimental animal studies before designing or starting a new animal experiment or clinical trial.

An international initiative to register animal studies must be started. In addition, journal editors must accept publications with so-called negative and neutral results, and promote data sharing.

Funding agencies should stimulate and fund systematic reviews. A recent article on forbes.com estimates that some major drug companies spend between US$4–$US11 billion per drug, once failure rates are factored in. Systematic reviews disclose inadequacies in methodology of individual studies. This helps improve future study design, and reduce failure rate of animal studies of new drugs [23].

Specifically, funding agencies can mandate systematic reviews of animal experiments as part of a funding. This will make the choice of animal models more evidence-based and provide better protection for human patients.

From a societal, ethical, and scientific perspective, laboratory animals must be used more efficiently. Better training of scientists who intend to work with animals, and improved research methods will assist the translational process, help the economic and ethical aspects of animal studies, and promote better health care and improved safety for patients and research participants.

Achieving success will depend upon co-operation between scientific journals, authors, animal research regulators, funding bodies, and peer reviewers. Progress also depends on the clinical research community demanding better quality pre-clinical research, and finding ways to improve communication with animal researchers whose pre-clinical work they may rely on [3].

Finally, a big step forward would be for organizations focused on systematic reviews of animal data, such as SYRCLE and CAMARADES, to join the worldwide network of the Cochrane Collaboration to improve links and cross-fertilisation between preclinical and clinical research, and facilitate translation from “bench to bedside.”
